# Do bleaching gels supplied in attachable syringes maintain their bleaching efficacy 72 hours after mixing?

**DOI:** 10.1007/s00784-026-07083-4

**Published:** 2026-08-18

**Authors:** Gabrielle Gomes Centenaro, Deisy Cristina Ferreira Cordeiro, Laryssa Mylenna Madruga Barbosa, Michael Willian Favoreto, Tobia Gaston Navarro, Fernanda Vieira Garrido, Alessandro D. Loguercio

**Affiliations:** 1 Department of Restorative Dentistry, School of Dentistry, State University of Ponta Grossa, Rua Carlos Cavalcanti, 4748 Bloco M, Sala 64-A, Uvaranas, Ponta Grossa, PR 84030-900 Brazil; 2 IDIBO Research Group, Rey Juan Carlos University, Alcorcón, Madrid Spain; 3 Department of Restorative Dentistry, University of São Paulo, São Paulo, SP Brazil

**Keywords:** Hydrogen Peroxide, Dental Enamel Permeability, Tooth Bleaching, Tooth permeability

## Abstract

**Objective:**

Evaluate bleaching efficacy (BE), hydrogen peroxide (HP) penetration into the pulp chamber, and selected physicochemical properties (HP concentration, pH, and viscosity) of in-office bleaching agents supplied in attachable syringes from different commercial brands, assessed immediately, after mixing and after 72 h of storage.

**Materials and methods:**

150 human premolars were allocated into fifteen groups (*n* = 10) according to (1) the commercial brand: DSP 35%, Nano White 35%, SSWhite 35%, Total Blanc 35%, HP Blue 35%, Potenza Bianco 38%, and Opalescence 40%; and (2) storage interval: immediate use or 72 h after mixing. One group stored in artificial saliva served as the negative control (no bleaching). HP concentration at both storage intervals was determined by titration, pH using a digital pH meter, and viscosity by rheometer. BE (ΔE_ab_, ΔE_00_ and ΔWI_D_) was measured with a digital spectrophotometer, and HP penetration (µg/mL) by UV–Vis spectrophotometry. Data were analyzed using ANOVA, Tukey’s, and Dunnett’s (α = 0.05).

**Results:**

HP concentration and pH differed among brands and decreased after storage for most gels. All gels produced significantly greater BE than the control (*p* < 0.05*)*. Storage time had minimal influence on BE. HP diffusion was material-dependent; Viscosity varied among brands, although most gels demonstrated shear-thinning behavior and storage-related changes.

**Conclusion:**

Gels showed material-dependent differences in properties, BE, and PH penetration, with minimal 72-hour storage effects on color. However, storage altered HP concentration, pH, and viscosity in some materials.

**Clinical Relevance:**

Evaluating gels after 72 h may supports safe reuse, maintaining efficacy while reducing material waste.

## Introduction

In recent years, the demand for esthetic dentistry has increased significantly [1] reflecting the growing influence of physical appearance on self-esteem, social interactions, and overall quality of life [2]. Among the available esthetic procedures, tooth bleaching stands out as one of the most requested treatments due to its minimally invasive nature, cost-effectiveness, and ability to provide immediate improvement in tooth color [3]. Consequently, bleaching procedures have become an integral part of daily dental practice, contributing to both esthetic outcomes and patient satisfaction [1, 4].

Tooth bleaching can be performed using different approaches, including at-home techniques that employ lower concentrations of hydrogen peroxide (HP) or carbamide peroxide (CP), as well as in-office procedures that typically use higher concentrations of HP (approximately 35% to 40%) [5, 6]. Bleaching gels at these concentrations are commercially available in three main presentations: self-mixing syringes, attachable syringes, and two-bottles systems [6]. While self-mixing gels and two-bottle systems allow use across multiple patients, attachable syringes are intended for single-patient application. In this format, HP and the thickening agent are combined immediately before the in-office procedure, preventing reuse for subsequent patients despite the considerable amount of residual bleaching gel remaining in the syringe [7, 8].

This limitation has important clinical and economic implications. Because the excess bleaching gel cannot be reused, a substantial portion of the material is often discarded (approximately 50%) [8], increasing treatment costs and reducing cost-effectiveness for dental practitioners. In contrast, presentations such as self-mixing gels or two-bottle systems offer greater flexibility and efficiency by allowing controlled dosing and use across multiple patients [9]. Therefore, the choice of gel presentation not only influences clinical handling but also affects the sustainability and practicality of in-office bleaching procedures.

Given the growing clinical and economic impact of bleaching gel waste, determining whether the material maintains its stability and efficacy after initial manipulation is highly relevant. Although attachable syringe systems are designed for single-use applications, the substantial amount of residual gel remaining after treatment raises questions regarding the feasibility of reuse within short storage intervals [8]. Investigating the chemical stability, pH behavior, and bleaching performance of previously mixed gels may provide valuable insights into optimizing clinical resources without compromising patient safety or treatment outcomes.

Consequently, studies exploring the reuse potential of in-office bleaching gels may contribute to the development of more sustainable and cost-effective whitening protocols in modern dental practice, particularly in scenarios involving short storage intervals or delayed use. From a clinical perspective, a new bleaching session may be performed after 72 h without increasing the tooth sensitivity [10], supporting the relevance of this evaluation period. This interval was selected because patients typically report tooth sensitivity for up to 48 h after bleaching procedures [3, 5, 6, 10]. Therefore, residual bleaching gel remaining in the syringe could potentially be reused after this period, either for the same patient or, under strict biosafety protocols, including replacement of the dispensing tip, for a different patient.

Therefore, the objective of this in vitro study was to evaluate bleaching efficacy, HP penetration into the pulp chamber, and the physicochemical properties of bleaching gels supplied in attachable syringes immediately after mixing and after 72 h of storage. The null hypotheses tested were that (1) no difference would be observed in bleaching efficacy (2) no difference would be detected in HP penetration into the pulp chamber, and (3) no difference would occur in the physicochemical properties of the bleaching gels between the two storage intervals.

## Materials and methods

### Ethical approval and criteria for teeth selection

This in vitro study was approved by the Research Ethics Committee of the State University of Ponta Grossa (Ponta Grossa, PR, Brazil), under protocol number (#7.204.932). A total of 150 healthy human maxillary premolars were obtained from the Human Teeth Bank. Teeth were examined under stereomicroscopic at 10× magnification (Lambda LEB-3, ATTO instruments, Hong Kong, China) to detect morphological defects or enamel cracks, which constituted exclusion criteria. Teeth presenting a Whiteness Index for Dentistry (WI_D_) value greater than 20, measured using a digital spectrophotometer (VITA Easyshade Advance 4.0, VITA Zahnfabrik, Bad Säckingen, Germany), were also excluded.

### Sample size calculation

The primary outcome was the color change (ΔE_00_) after in-office bleaching with attachable syringe systems. Based on a previous in vitro study [11], assuming a standard deviation of 1.1 and an equivalence limit of ΔE_00_ = 1.8 (clinically acceptable bleaching threshold) [12], with α = 5% and 90% power, nine specimens per group was required. One additional tooth per group was included to compensate for potential losses. Therefore, 10 teeth were allocated to each of the 15 experimental groups, totaling 150 teeth.

### Experimental design

Teeth were randomly assigned to 15 groups (*n* = 10). All specimens, including the negative control group, were stored in artificial saliva (Efficacy Pharmacy, Ponta Grossa, PR, Brazil) composed of carboxymethylcellulose, sodium chloride, potassium chloride, magnesium chloride, dibasic calcium phosphate, glycerin, xylitol, and distilled water [13], throughout the study period. The artificial saliva was replaced daily to better simulate the oral environment. The negative control remained under the same storage conditions but was not exposed to any bleaching protocol.

The remaining groups were distributed according to the commercial brands of in-office bleaching gels in attachable syringe format: DSP White Clinic 35% Calcium (DW; DSP Biomedical Group, Campo Largo, PR, Brazil); Nano White 35% (NW; DMC, São Paulo, SP, Brazil); SSWhite 35% (SS; Duflex, Juiz de Fora, MG, Brazil); Total Blanc One-Step 35% (TS; DFL, Rio de Janeiro, RJ, Brazil); Whiteness HP Blue 35% (WB; FGM Dental Group, Joinville, SC, Brazil); Potenza Bianco Pro SS 38% (PB; PHS Group, Joinville, SC, Brazil), and Opalescence XTra Boost 40% (OB; Ultradent, South Jordan, UT, USA) (Table 1).

**Table 1:** Commercial bleaching gel used in each group (batch number and composition)

Product (Batch number)	Composition	Application
DSP White Clinic 35% Calcium (DW) (29220241)	35% hydrogen peroxide, thickeners, neutralizers, coloring mix, glycols, deionized water, calcium.	Mix the two phases of the gel with the syringes connected, pushing the plungers eight times on each side (total of 16 times). The gel application period was 40 min, two sessions.
Nano White 35% (NW) (15735)	35% hydrogen peroxide, thickeners, neutralizers, glycols, deionized water.	Mix the two phases of the gel with the syringes connected, pushing the plungers two times on each side (total of 4 times). The gel application period was 40 min, two sessions.
SS White 35% (SS) (311223)	35% hydrogen peroxide, thickeners, neutralizers, glycols, deionized water.	Mix the two phases of the gel with the syringes connected, pushing the plungers five times on each side (total of 10 times). The gel application period was 40 min, two sessions.
Total Blanc One-Step 35% (TS) (440824)	35% Hydrogen peroxide, thickener, water, vegetable extracts, sequestrating agents, amide, colorants and glycol	Mix the two phases of the gel with the syringes connected, pushing the plungers seven times on each side (total of 14 times). The gel application period was 40 min, two sessions.
Whiteness HP Blue 35% (WB) (150925)	35% hydrogen peroxide, deionized water, thickeners, violet colorant, glycol, neutralized agents and desensitizing agents, 3% calcium gluconate.	Mix the two phases of the gel with the syringes connected, pushing the plungers four times on each side (total of 8 times). The gel application period was 40 min, two sessions.
Potenza Bianco Pro SS 38% (PB) (06072023-33666)	38% hydrogen peroxide, thickeners, neutralizers, colorant and deionized water.	Mix the two phases of the gel with the syringes connected, pushing the plungers ten times on each side (total of 20 times). The gel application period was 40 min, two sessions.
Opalescence XTra Boost 40% (OB) (D0QW1)	40% hydrogen peroxide, thickeners, pH regulators, 1.1% sodium fluoride, 3% potassium nitrate.	Mix the two phases of the gel with the syringes connected, pushing the plungers twenty-five times on each side (total of 50 times). The gel application period was 40 min, two sessions.

**Fig. 1:**
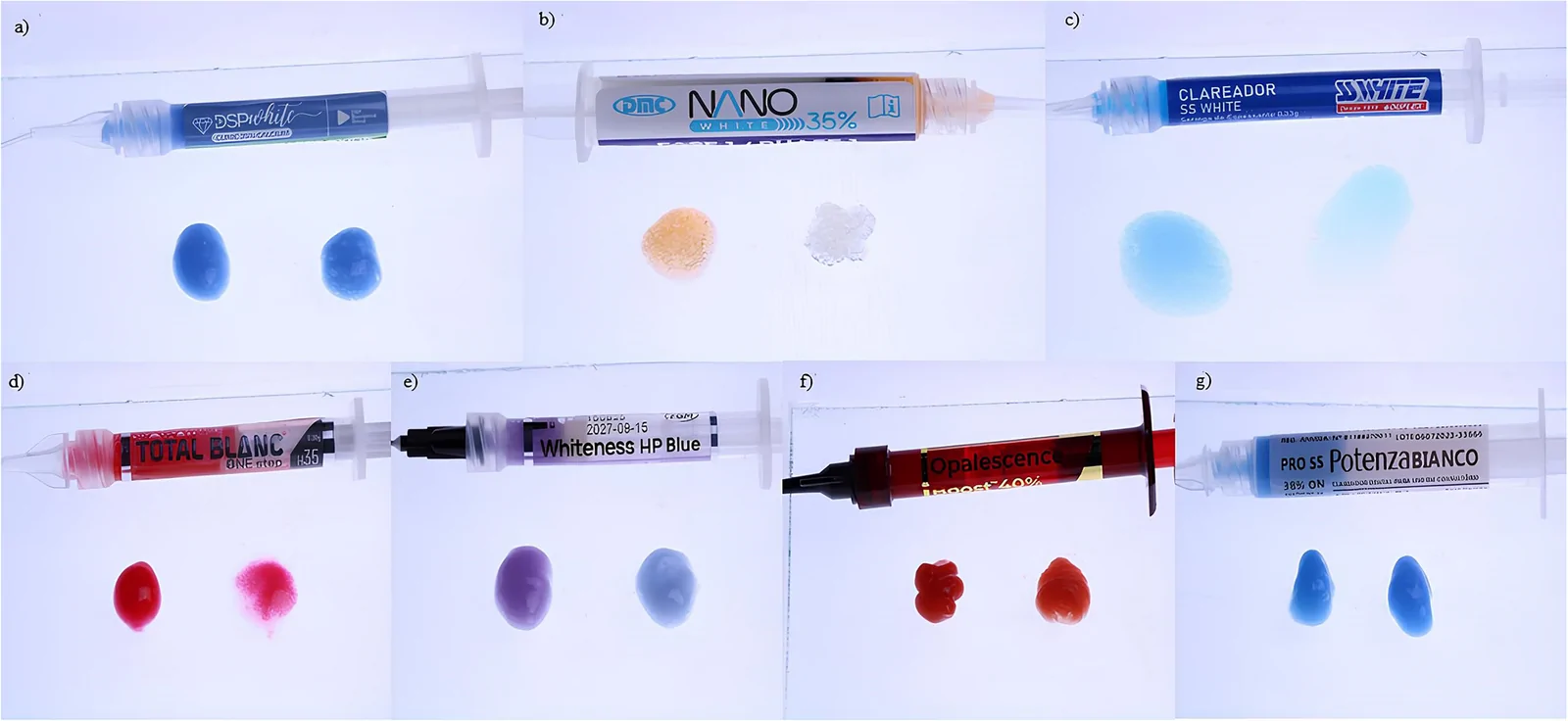
In each photo (**a** to **g**), immediately after mixing for each commercial brand of attachable syringes is shown on the left, and 72 hours after mixing and storage is shown on the right. In ’a’DSP White Clinic 35% Calcium (DW), in ’b’ Nano White 35% (NW), in ’c’ SS White 35% (SS), in ‘d’ Total Blanc One-Step 35% (TS), in ’e’ Whiteness HP Blue 35% (WB), in ’f’ Potenza Bianco Pro SS 38% (PB) and in ’g’ Opalescence Xtra Boost 40% (OB)

For each bleaching gel, two storage intervals were evaluated: immediately after mixing and 72 h of refrigerated storage. The 72-hour interval was selected because previous clinical evidence has shown that a new in-office bleaching session may be performed after 48 h without increasing tooth sensitivity [10], making this a clinically relevant period for evaluating the potential reuse of bleaching gels (Fig. 1).

### Measurement of initial HP concentration of the bleaching gels

Attachable syringes from all commercial brands were subjected to titration with standardized potassium permanganate solution immediately and 72 h after mixing HP and the thickener [8]. The potassium permanganate solution (0.02 mol/L) was standardized using sodium oxalate. At each time point, approximately 0.01 g of bleaching gel was weighed using an analytical balance (AUX220, Shimadzu, Kyoto, Japan), diluted in 20 mL of ultrapure water, and mixed with 20 mL of 1.0 mol/L sulfuric acid. HP concentration was determined by oxidation-reduction titration with 0.02 mol/L potassium permanganate [11], added at 0.1 mL/s until a persistent violet coloration indicated the equivalence point, which means that all the HP was consumed. Measured HP concentrations were compared with manufacturer-declared values and evaluated for stability after 72 h of refrigerated storage.

### Ph measurements

The pH of each bleaching gel was measured immediately after mixing and after 72 h using a digital pH meter equipped with a microelectrode (Extech pH 100: ExStik pH Meter; Extech instruments, Nashua, NH, USA), following a previously established method [13]. The device was calibrated using standard buffer solutions at pH 4.0 and 7.0 prior to measurements. All measurements were performed in triplicate by placing the bleaching gel in direct contact with the pH electrode at both time points. The obtained values were also compared with those reported by the manufacturer for the bleaching gels immediately after mixing [13]. The pH values were classified as acidic (ranging from 0 to 6), neutral (7), and alkaline (ranging from 8 to 14).

### Viscosity of the bleaching gel

The viscosity was assessed as a function of shear rate using a controlled-strain rheometer (DHR-2, TA Instruments, Houston, TX, USA) equipped with a 2° cone-and-plate geometry (40 mm in diameter). Temperature control was maintained at 37 °C using a Peltier system integrated into the rheometer to simulate oral conditions. Measurements were performed immediately and 72 h after mixing. To evaluate thixotropic behavior, a continuous shear flow test was conducted for 40 min at a constant shear rate of 5 s⁻¹. Sample temperature was maintained throughout the test using the integrated heating/cooling system. All procedures followed manufacturer instructions and as previously described protocols [8].

### Sample preparation

The roots of the premolars were sectioned approximately three mm apical to the cementoenamel junction using a low-speed diamond disc (Isomet 1000, Buehler Ltd., Lake Bluff, IL, USA). The pulp tissue was carefully removed, and the pulp chamber was rinsed with deionized water. Access to the pulp chamber was enlarged using a #1014 spherical bur (KG Sorensen, Barueri, SP, Brazil), while avoiding contact with the internal buccal wall. This procedure enabled the introduction of 25 µL of buffer solution into the pulp chamber using a micropipette (LABMATE Soft, HTL Lab Solutions, Warsaw, Poland) [11, 13].

### Evaluation of the specimen thickness

After preparation, periapical radiographs were obtained using a Timex 70 C X-ray unit (Gnatus, Ribeirão Preto, SP, Brazil) [8]. The mesial surface of each tooth was positioned in contact with the radiographic film. A standardized exposure time of 0.5 s and a 30-cm focus-object distance were used. The central X-ray beam (70 kVp; 7 mA) was directed at a 90° angle to the distal surface of the tooth. Radiographic images were digitally acquired using the New IDA software (Dabi Atlante, Ribeirão Preto, SP, Brazil). Teeth were included only if the combined enamel and dentin thickness ranged from 2.5 to 3.5 mm [8, 11, 14].

### Initial color evaluation

Individual positioning guides were fabricated for each specimen using the same green putty-consistency vinyl polysiloxane material (Coltoflax, Coltene, Altstätten, Switzerland) throughout the study to standardize spectrophotometer positioning and minimize any potential influence of guide color on the measurements [15]. A 6-mm-diameter circular window was created in the middle third of the buccal surface using a custom metallic device to ensure reproducible positioning of the spectrophotometer tip (VITA Easyshade Advance 4.0, VITA Zahnfabrik, Bad Säckingen, Germany) [11, 16]. All color measurements were performed in a room under controlled artificial lighting to minimize interference from ambient and natural light. The spectrophotometer tip was inserted into the positioning guide, and the color coordinates (L*, a* and b*) were recorded. The L* coordinate represents lightness (0 = black; 100 = white), a* represents the red-green axis, and b* represents the yellow-blue axis [17].

### Treatment protocols

All bleaching procedures were performed by a single calibrated and experienced operator. Within each evaluation time, the order of specimen bleaching was randomized using the Sealed Envelope online randomization tool (https://www.sealedenvelope.com), and specimens from the different experimental groups were randomly allocated to the bleaching sessions, rather than processed group by group, thereby minimizing potential operator- and time-related bias.

Specimens were positioned with the occlusal surface in contact with a wax plate to allow access to the pulp chamber. The buccal surface was isolated using a light-cured resin barrier (Top Dam, FGM Dental Group, Joinville, SC, Brazil), to delimit a standardized 6 × 6 mm treatment area [11]. The area was marked using a dry-point compass with standardized measurements. In the experimental groups, in-office bleaching gels supplied in attachable syringe were applied according to the manufacturers’ instructions (Table 1) to the buccal enamel surface, considering the assigned storage interval. The bleaching gel was applied to completely cover the standardized area of the teeth to be bleached.

The application time was standardized to 40 min per session (within the manufacturer-recommended 30–50 min range) to ensure comparability across groups. Each specimen received two bleaching sessions performed at a one-week interval. Depending on the experimental group, the bleaching gel was applied either immediately after mixing (0 h) or 72 h of refrigeration storage [18]. Immediately after mixing, the bleaching gels assigned to the 72-hour groups were stored under refrigeration (4–8 °C) for 72 h before application [17]. Refrigeration was performed only after mixing, whereas the manufacturer recommends immediate use of the bleaching gel after preparation. This storage protocol was adopted to evaluate the physicochemical stability and potential reuse of the residual bleaching gel after short-term refrigerated storage. After each bleaching session, the gel was carefully removed with gauze, and the buccal surface was rinsed with deionized water. The control group received no bleaching treatment. During the experimental period, all specimens, including the control group, were stored in artificial saliva, with daily replacement at 37 °C, as previously described [13].

### Analytical curve development for HP measurements

All reagents were analytical grade and used without further purification. Solutions were prepared with deionized water. An analytical calibration curve was established from a 5000 µg/mL stock solution prepared from 50% HP (LABSYNTH, Diadema, SP, Brazil). The stock solution was diluted in acetate buffer solution (pH = 4) and titrated with potassium permanganate solution to determine the analytical grade and actual concentration, as previously described protocols [8, 11, 13]. Based on this value, serial dilutions ranging from 0.000 to 0.404 µg/mL were prepared to construct the calibration curve. Known concentrations of HP were measured using a Cary UV-Vis 100 spectrophotometer (Varian, Palo Alto, CA, USA) [11].

### Penetration of HP into the pulp chamber

Before the bleaching procedures, a 25 µL aliquot of acetate buffer solution (pH = 4) was introduced into the pulp chamber of each tooth to absorb and stabilize any HP that penetrated during bleaching [8, 11, 13]. Immediately after each bleaching session, the acetate buffer solution was collected from the pulp chamber and transferred to a glass tube [11].

To ensure complete recovery of penetrated HP, the pulp chamber was rinsed four additional times with 25 µL of acetate buffer, and each rinse was added to the same tube. Sequentially, 100 µL of 0.5 mg/mL of leucocristal Violeta (Sigma Chemical Co., St Louis, MO, USA) and 50 µL of 1 mg/mL horseradish peroxidase enzyme (Peroxidase Type VI-A, Sigma Cemical Co., St. Louis, MO, USA) were added, followed by 2.725 µL of deionized water.

This procedure was performed for each specimen at both evaluation time points. The final solution was analyzed using a Cary 1000 UV-Vis spectrophotometer (Varian, Palo Alto, CA, USA). According to Beer-Lambert’s law, HP concentration (µg/mL) was determined based on absorbance values and comparison with a previously established calibration curve [8, 11, 13].

### Final color evaluation

Bleaching efficacy was assessed before any procedure (baseline) and one week after completion of the treatment protocol using a digital spectrophotometer (VITA Easyshade Advance 4.0, VITA Zahnfabrik, Bad Säckingen, Germany). The same color parameters (L*, a*, and b*) previously mentioned were recorded.

Color change was calculated using the CIELab formula (L’Eclairage, 1978): ∆E_ab_ = [(∆L*)^2^ + (∆a*)^2^ + (∆b*)^2^]^1/2^, and the CIEDE 2000 formula [17]: ∆E_00_ = [(ΔL’/k_L_S_L_)^2^ + (ΔC’/k_C_S_C_)^2^ + (ΔH’/k_H_S_H_)^2^ + RT (ΔC’/k_C_S_C_) (ΔH’/ k_H_S_H_)]^1/2^ [19]. The Whiteness Index for Dentistry (WI_D_) was calculated as [12]: WI_D_=0.551×L − 2.324×a − 1.1×b.

Changes in WI_D_ (ΔWI_D_) were determined by subtracting baseline values from post-treatment values. Color differences were considered clinically relevant when exceeding the 50:50% acceptability threshold: 2.7 for ∆E_ab_, 1.8 for ∆E_00_, and 2.6 for ∆WI_D_ [20, 21].

This Whiteness Index for Dentistry (ΔWI_D_) was specifically developed to quantify tooth whiteness based on the CIELAB coordinates. ΔWI_D_ places greater weighting on variations in lightness (L*) and the blue–yellow axis (b*), which are the principal determinants of perceived tooth whitening. Consequently, this parameter demonstrates superior sensitivity to bleaching-induced changes and exhibits a stronger correlation with visual assessments of tooth whiteness when compared to ΔE-based metrics [12].

### Statistical analysis

Data distribution was assessed using the Kolmogorov–Smirnov test, and homogeneity of variances was verified using Barlett test (data not shown). As normality and homoscedasticity of variances were confirmed, bleaching efficacy (ΔE_ab_, ΔE_00_ and ∆WI_D_), HP concentration (µg/mL), and pH were analysed using two-way ANOVA (bleaching gels × time), followed by Tukey’s post hoc test for pairwise comparisons among time points. Additionally, Dunnett’s test was applied to compare each bleaching gel with the control group (α = 0.05). The viscosity results were analyzed descriptively. Statistical significance was set at α = 0.05 for all analyses (IBM SPSS version 22.0, SPSS, Chicago, IL, USA).

## Results

### HP concentration

Table 2 presents the HP concentrations (%) and pH values of the commercial attachable-syringe bleaching gels evaluated in this study. Significant differences were observed among the bleaching gels and between storage times (immediately after mixing and 72 h later) (Table 2; *p* < 0.05).

**Table 2:** Means (± standard deviations) of the concentration (%) and pH, measured immediately after mixing and after 72 h of storage among the experimental groups

Experimental groups	Concentration (%)		pH	
	Immediately after mixing	72 h after mixing	Immediately after mixing	72 h after mixing
DSP White Clinic 35% Calcium (DW)	33.0 ± 0.6 B	30.5 ± 0.1 C	5.9 ± 0.1 C	5.8 ± 0.1 C
Nano White 35% (NW)	33.0 ± 0.5 B	31.6 ± 0.1 C	6.9 ± 0.3 B	6.2 ± 0.1 C
SS White 35% (SS)	20.3 ± 0.1 E	20.0 ± 0.1 E	6.5 ± 0.1 B	5.0 ± 0.1 C
Total Blanc One-Step 35% (TS)	34.3 ± 0.9 B	30.3 ± 0.2 C	7.3 ± 0.3 B	5.1 ± 0.1 C
Whiteness HP Blue 35% (WB)	34.2 ± 0.5 B	27.8 ± 0.1 D	9.0 ± 0.2 A	7.7 ± 0.3 B
Potenza Bianco Pro SS 38% (PB)	38.6 ± 0.4 A	34.7 ± 0.1 B	4.8 ± 0.1 D	4.2 ± 0.1 D
Opalescence Xtra Boost 40% (OB)	38.1 ± 0.1 A	37.0 ± 0.1 A	7.7 ± 0.3 D	7.1 ± 0.3 B

(*) Means followed by the same letters (capital) indicate no statistically significant differences among groups for each property (Tukey’s post-hoc test, *p* < 0.05)

At baseline (immediately after mixing), all bleaching gels presented HP concentrations within the acceptable range relative to their manufacturer-declared nominal values, according to the ISO [22] tolerance limits (− 30% to + 10%), with the exception of SS, which showed a substantially lower HP concentration than expected for a product labelled as 35% HP (Table 2).

After 72 h of refrigerated storage following mixing, a reduction in HP concentration was observed for most bleaching gels. WB exhibited the greatest decrease, whereas SS showed the smallest reduction. NW and OB also showed minimal changes, while the remaining bleaching gels presented intermediate reductions during the evaluated storage period (Table 2).

### pH measurements

Also in Table 2, regarding pH at baseline (immediately after mixing), WB showed the highest values, characterizing an alkaline pH, and differed significantly from all other bleaching gels (*p* < 0.05). NW, SS, TS, and OB presented near-neutral pH values, whereas PB showed the lowest pH, indicating the most acidic formulation. DW also exhibited an acidic pH, although less pronounced than PB.

Over time, OB maintained a stable pH, with no significant differences between the evaluated time points (*p* > 0.05). The bleaching gels with initially neutral pH (NW, SS, and TS) showed a reduction in pH after 72 h, becoming more acidic. The initially alkaline gel (WB) also exhibited a decrease in pH over time, reaching near-neutral values after storage. In contrast, the acidic gels (PB and DW) maintained their low pH throughout the evaluated period, with no statistically significant changes between time points (*p* > 0.05).

### Bleaching efficacy evaluation

Table 3 shows the bleaching efficacy measurements. No significant differences were observed when baseline WI_D_ values among groups were evaluated (Table 3; *p* > 0.05). Regarding bleaching efficacy, a significant difference was found when comparing the control group with all experimental groups (*p* < 0.05 for ΔE_ab_, ΔE_00_, and ΔWI_D_; Table 3).

**Table 3:** Means (± standard deviations) of the color change in different objective assessments (ΔE_ab_, ΔE_00_ and ΔWI_D_) among the experimental groups

In-office bleaching gels	Time	Color parameters (*)		
		ΔE_ab_	ΔE_00_	ΔWI_D_
DSP White Clinic 35% Calcium (DW)	Immediately after mixing	7.9 ± 1.7 B	4.7 ± 1.1 B	6.4 ± 3.0 C
	72 h after mixing	8.3 ± 2.5 A, B	4.6 ± 1.5 B	7.2 ± 3.3 C
Nano White 35% (NW)	Immediately after mixing	9.7 ± 2.7 A	6.1 ± 1.8 A, B	9.2 ± 4.1 A
	72 h after mixing	8.9 ± 2.4 A, B	4.7 ± 1.6 B	6.9 ± 1.7 C
SS White 35% (SS)	Immediately after mixing	10.2 ± 3.7 A	5.7 ± 2.5 A, B	8.9 ± 4.1 A, B
	72 h after mixing	8.8 ± 2.3 A, B	4.8 ± 1.4 B	7.4 ± 3.2 C, B
Total Blanc One-Step 35% (TS)	Immediately after mixing	12.5 ± 5.4 A	7.8 ± 3.6 A	11.4 ± 5.2 A
	72 h after mixing	8.3 ± 2.5 A, B	4.8 ± 1.6 B	8.1 ± 2.7 A, B
Whiteness HP Blue 35% (WB)	Immediately after mixing	10.1 ± 3.5 A	6.6 ± 2.0 A	10.5 ± 3.0 A
	72 h after mixing	9.4 ± 2.0 A, B	5.2 ± 1.3 A, B	8.7 ± 2.8 A, B
Potenza Bianco Pro SS 38% (PB)	Immediately after mixing	7.0 ± 2.8 B	4.1 ± 1.6 C	6.6 ± 3.4 C
	72 h after mixing	8.0 ± 3.4 B	4.4 ± 2.1 C	6.6 ± 3.2 C
Opalescence Xtra Boost 40% (OB)	Immediately after mixing	11.8 ± 2.9 A	7.4 ± 1.9 A	12.2 ± 2.7 A
	72 h after mixing	11.8 ± 3.0 A	6.7 ± 2.1 A	11.4 ± 2.2 A

(*) Means followed by the same letters no statistically significant differences among groups for each color parameter (Tukey’s post-hoc test, *p* < 0.05)

For ΔE_ab_, OB exhibited the highest color change. At the immediate time point, PB and DW showed the lowest values, differing from the other bleaching gels. After 72 h of storage, DW no longer differed from the higher-performing groups, whereas PB remained among the gels with lower ΔE_ab_ values. The remaining materials (NW, SS, TS, and WB) presented intermediate results (Table 3). A similar pattern was observed for ΔE_00_, with OB maintaining the highest values and PB among the lowest, while the other gels showed intermediate bleaching efficacy. For ΔWI_D_, OB again demonstrated the highest values, whereas PB and DW exhibited the lowest color change, and NW, SS, TS, and WB presented intermediate results (Table 3).

When the same bleaching gel was compared across storage times (immediate vs. 72 h after mixing), a significant difference was observed only for TS in ΔE₀₀, and for NW in ΔWI_D_, both showing reduced color change after storage (*p* < 0.05). No other significant differences over time were observed for the remaining groups (Table 3).

### Amount of HP into the pulp chamber

The specimens exhibited standardized thicknesses, with values varying from 2.8 to 3.5 mm (data not shown, *p* = 0.65). Table 4 shows the amount of HP detected into the pulp chamber. In DW, NW, SS and OB groups, the amount of HP in the pulp chamber did not differ between the immediate and 72 h storage conditions, with the OB group demonstrating the lowest values among these groups. The TS and PB groups showed an increase in HP diffusion after 72 h, while WB group showed a decrease in HP values after 72 h of mixing.

**Table 4:** Means (± standard deviations) of the hydrogen peroxide concentration (µg/mL) detected into the pulp chamber among the experimental groups

In-office bleaching gels*	Time	Hydrogen peroxide concentration (µg/mL)
DSP White Clinic 35% Calcium (DW)	Immediately after mixing	0.48 ± 0.16 C
	72 h after mixing	0.49 ± 0.15 C
Nano White 35% (NW)	Immediately after mixing	0.30 ± 0.05 C
	72 h after mixing	0.39 ± 0.10 C
SS White 35% (SS)	Immediately after mixing	0.45 ± 0.11 C
	72 h after mixing	0.46 ± 0.08 C
Total Blanc One-Step 35% (TS)	Immediately after mixing	0.22 ± 0.11 B
	72 h after mixing	0.34 ± 0.08 C
Whiteness HP Blue 35% (WB)	Immediately after mixing	0.23 ± 0.06 B
	72 h after mixing	0.11 ± 0.05 A
Potenza Bianco Pro SS 38% (PB)	Immediately after mixing	0.49 ± 0.09 C
	72 h after mixing	0.80 ± 0.16 D
Opalescence Xtra Boost 40% (OB)	Immediately after mixing	0.24 ± 0.05 B
	72 h after mixing	0.22 ± 0.05 B
Control Group (CG)	-	0.001 ± 0.001**

(*) Means followed by the same letter indicate no statistically significant differences among groups (Tukey’s post-hoc test, *p* < 0.05)

(**) All experimental groups showed statistically significant differences compared with the control group (Dunnett’s post-hoc test, *p *< 0.05)

### Viscosity of the bleaching gel

The initial and final viscosities of the bleaching gels varied according to the commercial brand (Table 5). OB, WB, and PB exhibited the highest, whereas TS and SS showed the lowest viscosities among the evaluated bleaching materials. Immediately after mixing, most gels demonstrated a progressive reduction in viscosity during the 40-minute shear test, indicating shear-thinning behavior and time-dependent flow behaviors. OB, NW, and TS exhibited the most pronounced decreases in viscosity under shear stress, while PB exhibited the smallest reduction among the highly structured gels, suggesting greater resistance to structural breakdown during testing. WB also demonstrated a consistent viscosity decrease over time while maintaining a structured rheological profile. After 72 h of storage, inter-material differences persisted. OB and WB maintained high initial viscosities and continued to exhibit substantial viscosity reductions under shear, indicating preservation of their gel network structure. NW also maintained a marked time-dependent decrease in viscosity. In contrast, the remaining materials exhibited altered rheological behavior after storage, with changes in both their initial viscosities and shear-dependent flow patterns.

**Table 5:** Average viscosity (thixotropy, Pa·s) at the two storage times for different experimental groups (*)

In-office bleaching gels*	Time	η₀ (Pa·s)	η₂₄₀₀ (Pa·s)	Thixotropic index (%;*)
DSP White Clinic 35% Calcium (DW)	Immediately after mixing	77.0	17.0	+ 78
	72 h after mixing	15.0	18.0	–20
Nano White 35% (NW)	Immediately after mixing	136	14.0	+ 90
	72 h after mixing	286	17.0	+ 94
SS White 35% (SS)	Immediately after mixing	1.44	1.29	+ 10
	72 h after mixing	0.02	0.10	–400
Total Blanc One-Step 35% (TS)	Immediately after mixing	53.0	1.30	+ 98
	72 h after mixing	0.04	0.70	–1650
Whiteness HP Blue 35% (WB)	Immediately after mixing	84.0	11.0	+ 87
	72 h after mixing	77.0	15.0	+ 81
Potenza Bianco Pro SS 38% (PB)	Immediately after mixing	82.0	72.0	+ 12
	72 h after mixing	72.0	33.0	+ 54
Opalescence Xtra Boost 40% (OB)	Immediately after mixing	466	42.0	+ 91
	72 h after mixing	470	2.10	+ 100

(*) The thixotropic index was calculated based on the relative change between initial viscosity (η₀) and final viscosity (η₂₄₀₀) under shear

## Discussion

Recent clinical studies have shown that the volume of bleaching gel applied to the enamel surface does not significantly influence bleaching efficacy, as thin and uniform layers produce whitening outcomes comparable to larger volumes [23, 24]. Consequently, a considerable amount of bleaching gel frequently remains unused after clinical application [25], particularly in attachable syringe systems [7], raising concerns regarding material waste and the feasibility of reuse [26, 27]. The present study investigated whether in-office bleaching gels supplied in attachable syringes maintain their bleaching efficacy, HP permeability and physicochemical properties after 72 h of refrigerated storage following mixing. Although high-concentration HP formulations are widely used for in-office bleaching, evidence regarding their post-mixing stability and performance remains scarce [3, 28].

Regarding bleaching efficacy, all bleaching gels produced significantly greater color change than the control group, and a similar whitening performance was maintained after 72 h of refrigerated storage [29], confirming the first null hypothesis. However, bleaching efficacy varied among the commercial formulations evaluated. Although the available HP concentration ranged from 33% to 38% immediately after mixing and from 27.8 to 37% after 72 h of storage, (except for SS which contained 20% HP) [8, 11], these values alone do not explain the differences in bleaching efficacy among the evaluated products. Therefore, formulation-related characteristics, including pH and rheological behavior, should also be considered when interpreting whitening performance.

Among the evaluated products, DW (WI_D_), NW (WI_D_; only after 72 h) and PB (ΔE₀₀ and WI_D_) exhibited the lowest bleaching efficacy despite presenting intermediate HP concentrations and pH values. These findings indicate that whitening performance cannot be explained solely by HP concentration or pH, suggesting that additional formulation-related characteristics may also influence bleaching performance. In the case of PB, the bleaching gel exhibited the lowest pH values both immediately after mixing [11, 30] and after 72 h of storage. Previous studies have consistently reported that acidic bleaching gels tend to show lower bleaching efficacy [11, 30]. This may be explained by the dissociation of HP into free radicals, which occurs more readily under alkaline conditions, as the pKa of HP is approximately 11.5 [31]. Consequently, higher pH values favor the formation of reactive oxygen species capable of oxidizing organic chromogens within dental tissues, thereby improving bleaching efficacy [32, 33].

For DW and NW, the reasons for their relatively lower bleaching efficacy remain unclear. Nevertheless, the present findings are consistent with those reported by da Silva et al. [11]. Interestingly, NW exhibited lower whitening efficacy immediately after mixing but improved ΔWI_D_ values after 72 h of refrigerated storage. Although the exact mechanism remains unknown, commercial bleaching gels differ in stabilizers, catalysts, thickening agents, and buffering systems, all of which may influence HP stability after mixing. Because these formulation characteristics were not evaluated in the present study, any explanation remains speculative. Further investigations are therefore needed to determine whether formulation characteristics or storage-related changes are responsible for the bleaching performance in this material.

All bleaching gels allowed measurable HP diffusion into the pulp chamber, confirming that transdentinal HP penetration is an inherent aspect of in-office bleaching procedures, as previously observed [11, 30]. However, the magnitude of diffusion was influenced by both the material formulation and storage time, leading to the rejection of the second null hypothesis. Three bleaching gels (WB, TS and PB) showed significant differences in the amount of HP detected in the pulp chamber after 72 h compared with baseline values. For WB, HP diffusion decreased after 72 h of storage. This finding may be partially explained by the lower concentration of available HP (27.8%) together with its relatively higher pH, both of which have previously been associated with reduced HP diffusion through dental tissues [11, 13]. Although the formulation also contains calcium, its specific contribution to HP diffusion was not investigated in the present study and therefore cannot be confirmed.

The other two products (TS and PB) showed a significant increase in the amount of HP detected in the pulp chamber over time. In the case of TS, the marked reduction in pH (2.3 units) after 72 h, shifting from an initially alkaline value to a more acidic pH (5.1), may explain this behavior [31]. For PB, despite no significant change in pH over time, the product showed the lowest pH among all groups (pH = 4.2). The decomposition of HP can generate reactive oxygen species and acidic by-products, which may contribute to localized reductions in pH [26, 28]. Acidic conditions may enhance HP stability and diffusion through dental tissues, which could help explain the higher HP penetration observed in these groups [11, 13, 31]. In addition, TS showed the lowest viscosity among all evaluated materials, which may also have contributed to the increased amount of HP detected inside the pulp chamber [34–36]. Although the acidic pH values observed for PB and TS may have influenced HP diffusion, they remained within the pH range commonly reported for commercially available in-office bleaching gels [11, 30]. Therefore, pH alone is unlikely to explain enamel demineralization, as this process is also influenced by factors such as HP concentration, formulation, application time, and the presence of remineralizing components. Nevertheless, because the relationship between pH, mineral loss, and bleaching formulation is multifactorial, further studies evaluating enamel surface properties after refrigerated storage are warranted.

The present findings also demonstrate that the rheological behaviour of in-office bleaching gels is dependent on material formulation and may be change after post-mixing storage. Clinically, viscosity is important because it directly influences gel retention on the enamel surface [34–36]. Therefore, differences in rheological behavior may partially explain the distinct HP diffusion patterns observed among the evaluated products. Notably, OB and WB maintained high viscosity values and consistent shear-thinning behavior immediately after mixing and after 72 h of storage, suggesting greater physicochemical stability throughout the experimental period [8]. It is possible that formulation components, as in the case of OB and WB, including fluoride and calcium, contributed to the greater physicochemical stability observed for these products. However, this hypothesis requires confirmation in future studies specifically designed to evaluate the role of these components.

This study has several limitations that should be considered when interpreting the present findings. First, as an in vitro investigation, the experimental conditions cannot fully reproduce the complexity of the oral environment, including pulpal pressure, dentinal fluid movement, and patient-related variables that may influence HP diffusion and bleaching efficacy. Second, substrate-related properties, such as enamel microhardness and surface roughness, were not evaluated and deserve further investigation, particularly because acidic bleaching gels have previously been associated with changes in these properties [34, 35]. Finally, although the present study demonstrated formulation-dependent changes after refrigerated storage, the chemical degradation products generated during storage and the mechanisms responsible for these alterations were not investigated. Because the effects of delayed use were formulation-dependent, the reuse of bleaching gels after 72 h should be considered with caution. It should also be emphasized that all bleaching gels were stored under refrigerated conditions; therefore, the present findings cannot be extrapolated to different storage temperatures [18]. Finally, clinical studies are needed to determine whether the physicochemical changes observed after refrigerated storage translate into differences in tooth sensitivity, bleaching efficacy, and long-term clinical performance [36–38].

## Conclusion

In-office bleaching gels demonstrated pronounced formulation-dependent differences in bleaching efficacy, physicochemical properties and trans-enamel and dentin diffusion, highlighting the influence of formulation characteristics on product performance. Although 72-hour storage had minimal impact on color outcomes, it significantly affected key parameters such as hydrogen peroxide concentration, pH, and viscosity in several materials. These findings clearly indicate that storage conditions can compromise material stability and, consequently, affect both the safety and clinical effectiveness of bleaching procedures. Therefore, the use and potential reuse of bleaching gels must be guided by formulation-specific evidence.

## Data Availability

No datasets were generated or analysed during the current study.

## References

[1] Goettems ML, Fernandez MDS, Donassollo TA, Henn Donassollo S, Demarco FF (2021) Impact of tooth bleaching on oral health-related quality of life in adults: a triple-blind randomised clinical trial. J Dent 105:103564. 10.1016/j.jdent.2020.103564 33359042

[2] Bersezio C, Estay J, Jorquera G, Peña M, Araya C, Angel P, Fernández E (2019) Effectiveness of Dental Bleaching With 37.5% and 6% Hydrogen Peroxide and Its Effect on Quality of Life. Oper Dent 44(2):146–155. 10.2341/17-229-C 30517065

[3] de Geus JL, Wambier LM, Kossatz S, Loguercio AD, Reis A (2016) At-home vs in-office bleaching: a systematic review and meta-analysis. Oper Dent 41:341–356. 10.2341/15-287-LIT 27045285

[4] Bersezio C, Martín J, Mayer C, Rivera O, Estay J, Vernal R, Haidar ZS, Angel P, Oliveira OB Jr, Fernández E (2018) Quality of life and stability of tooth color change at three months after dental bleaching. Qual Life Res 27(12):3199–3207. 10.1007/s11136-018-1972-7 30132252

[5] Reis A, Mendonça da Silva L, Martins L, Loguercio A (2018) In-office tooth whitening. Clin Dent Ver 2:10. 10.1007/s41894-018-0021-9

[6] Kielbassa AM, Maier M, Gieren AK, Eliav E (2015) Tooth sensitivity during and after vital tooth bleaching: A systematic review on an unsolved problem. Quintessence Int 46(10):881–897. 10.3290/j.qi.a34700 26396993

[7] Bernardi LG, Favoreto MW, Carneiro TS, Mena-Serrano A, Borges CPF, Reis A, Loguercio AD (2022) Use of an applicator brush with high concentration bleaching gels. Clin Oral Investig 26:6387–6395. 10.1007/s00784-022-04594-8 35776203

[8] Forville H, Favoreto MW, Carneiro TS, Terra R, Pinheiro LA, Borges C, Loguercio AD, Reis A (2023) Bleaching gels used after 1 week of mixing: efficacy, hydrogen peroxide penetration, and physical-chemical properties. Oper Dent 48(5):564–574. 10.2341/23-010-L 37721110

[9] Marson FC, Gonçalves RS, Silva CO, Cintra LT, Pascotto RC, Santos PH, Briso AL (2015) Penetration of hydrogen peroxide and degradation rate of different bleaching products. Oper Dent 40:72–79. 10.2341/13-270-l 24828134

[10] de Paula EA, Nava JA, Rosso C, Benazzi CM, Fernandes KT, Kossatz S, Loguercio AD, Reis A (2015) In-office bleaching with a two- and seven-day intervals between clinical sessions: A randomized clinical trial on tooth sensitivity. J Dent 43(4):424–429. 10.1016/j.jdent.2014.09.009. Epub 2014 Sep 23. PMID: 25257824 25257824

[11] da Silva KL, Favoreto MW, Centenaro GG, Bernardi LG, Borges CPF, Reis A, Loguercio AD (2023) Can all highly concentrated in-office bleaching gels be used as a single-application? Clin Oral Investigs 27(7):3663–3671. 10.1007/s00784-023-04980-w 37017758

[12] Perez Mdel M, Ghinea R, Rivas MJ, Yebra A, Ionescu AM, Paravina RD, Herrera LJ (2016) Development of a customized whiteness index for dentistry based on CIELAB color space. Dent Mater 32:461–467. 10.1016/j.dental.2015.12.008 26778404

[13] Carneiro TS, Favoreto MW, Bernardi LG, Bandeca MC, Borges C, Reis A, Loguercio AD (2023) Application tip and concentration of a self-mixing bleach: hydrogen peroxide inside the pulp chamber, color change, and amount of bleaching gel used. Oper Dent 8(2):146–154. 10.2341/21-053-L 36786759

[14] Barbosa LMM, de Souza Carneiro T, Favoreto MW, Borges CPF, Reis A, Meireles SS, Loguercio AD (2024) Whitening toothpastes with hydrogen peroxide concentrations vs. at-home bleaching. Clin Oral Investig 28(8):436. 10.1007/s00784-024-05823-y 39030259

[15] Santana TR, Silva PFD, Santana MLC, de Mattos CLLB, Faria-E-Silva AL (2024) Influence of repositioning guides’ color and usage on precision in tooth color measurement with a clinical spectrophotometer. J Appl Oral Sci 22(32):e20230348. 10.1590/1678-7757-2023-0348 PMC11018295 38537029

[16] CIE (1977) Recommendations on uniform color spaces, color difference equations, and metric color terms. Color Res Appl 2:5–6. 10.1002/j.1520-6378.1977.tb00102.x

[17] Chisini LA, Conde MCM, Meireles SS, Dantas RVF, Sarmento HR, Della Bona Á, Corrêa MB, Demarco FF (2019) Effect of temperature and storage time on dental bleaching effectiveness. J Esthet Restor Dent 31(1):93–97. 10.1111/jerd.12439 30379397

[18] Cordeiro DCF, Favoreto MW, Centenaro GG, Gumy FN, Loguercio AD, Borges CPF, Reis A (2024) At-home bleaching with carbamide peroxide with concentrations below 10%: bleaching efficacy and permeability in the pulp chamber. Clin Oral Investig 28:224. 10.1007/s00784-024-05604-7 38509406

[19] Luo MR, Cui G, Rigg BJC (2001) The development of the CIE 2000 colour-difference formula: CIEDE2000. Color Res Appl 26:340–350. 10.1002/col.1049

[20] Paravina RD, Ghinea R, Herrera LJ, Bona AD, Igiel C, Linninger M, Sakai M, Takahashi H, Tashkandi E, Perez Mdel M (2015) Color difference thresholds in dentistry. J Esthet Restor Dent 27(Suppl 1):S1–S9. 10.1111/jerd.12149 25886208

[21] Pérez MM, Herrera LJ, Carrillo F, Pecho OE, Dudea D, Gasparik C, Ghinea R, Bona AD (2019) Whiteness difference thresholds in dentistry. Dent Mater 35:292–297. 10.1016/j.dental.2018.11.022 30527588

[22] ISO 28399 2021 I dentistry - external tooth bleaching products

[23] Centenaro GG, Favoreto MW, Cordeiro DCF, Gumy FN, Machado AG, Cochinski GD, Reis A, Loguercio AD (2024) Effect of the type of application tip for 35% hydrogen peroxide on bleaching efficacy and tooth sensitivity: a randomized clinical trial. J Esthet Restor Dent 36(7):1029–1037. 10.1111/jerd.13219 38475979

[24] Carneiro TS, Favoreto MW, Ferreira MWC, Bernardi LG, Andrade HF, Bandeca MC, Reis A, Ceballos García L, Loguercio AD (2023) In-office dental bleaching in adolescents using 6% hydrogen peroxide with different application tips: randomized clinical trial. J Appl Oral Sci 31:e20230216. 10.1590/1678-7757-2023-0216 37909527 PMC10609651

[25] Centenaro GG, Favoreto MW, Cordeiro DCF, Carneiro TS, Basting RT, Reis A, Loguercio AD (2024) Effect of a brush tip on in-office bleaching gels in an attachable syringe: hydrogen peroxide penetration, bleaching efficacy and amount of gel expended. J Dent 148:105239. 10.1016/j.jdent.2024.105239 39019248

[26] Joiner A (2006) The bleaching of teeth: a review of the literature. J Dent 34(7):412–419. 10.1016/j.jdent.2006.02.002 16569473

[27] Alqahtani MQ (2014) Tooth-bleaching procedures and their controversial effects: A literature review. Saudi Dent J 26(2):33–46. 10.1016/j.sdentj.2014.02.002 25408594 PMC4229680

[28] Sulieman M (2006) An overview of bleaching techniques: history, chemistry, safety and legal aspects (part 1). SADJ 61(7):304–312 17133792

[29] de Freitas IM, Carneiro TS, Forville H, Favoreto MW, Wendlinger M, Ñaupari-Villasante R, Gonçalves S, Vallejo-Izquierdo L, Loguercio AD (2025) Efficacy of In-office Bleaching Gel One Week After Mixing: A Randomized Clinical Trial. Oper Dent 50(4):358–368. 10.2341/24-115-C 40992750

[30] Gumy FN, da Silva KL, Gumy MN, Forville H, Cordeiro DCF, Favoreto MW, Loguercio AD, Reis A (2024) The decomposition rate and bleaching efficacy of in-office bleaching gels with different pHs: a randomized controlled trial. Clin Oral Investig 28(8):440. 10.1007/s00784-024-05821-0 39042288

[31] Torres CR, Crastechini E, Feitosa FA, Pucci CR, Borges AB (2014) Influence of pH on the effectiveness of hydrogen peroxide whitening. Oper Dent 39:E261–E268. 10.2341/13-214-l 25136903

[32] Kossatz S, Martins G, Loguercio AD, Reis A (2012) Tooth sensitivity and bleaching effectiveness associated with use of a calcium-containing in-office bleaching gel. J Am Dent Assoc 143(12):e81–e87. 10.14219/jada.archive.2012.0075 23204096

[33] Basting RT, Amaral FL, França FM, Flório FM (2012) Clinical comparative study of the effectiveness of and tooth sensitivity to 10% and 20% carbamide peroxide home-use and 35% and 38% hydrogen peroxide in-office bleaching materials containing desensitizing agents. Oper Dent 37(5):464–473. 10.2341/11-337-C 22616927

[34] Magalhães JG, Marimoto AR, Torres CR, Pagani C, Teixeira SC, Barcellos DC (2012) Microhardness change of enamel due to bleaching with in-office bleaching gels of different acidity. Acta Odontol Scand 70(2):122–126. 10.3109/00016357.2011.600704 21780968

[35] Borges AB, Yui KC, D’Avila TC, Takahashi CL, Torres CR, Borges AL (2010) Influence of remineralizing gels on bleached enamel microhardness in different time intervals. Oper Dent 35(2):180–186. 10.2341/09-117-L 20420061

[36] Mena-Serrano AP, Parreiras SO, do Nascimento EM, Borges CP, Berger SB, Loguercio AD, Reis A (2015) Effects of the concentration and composition of in-office bleaching gels on hydrogen peroxide penetration into the pulp chamber. Oper Dent 40:E76–82. 10.2341/13-352-25535786

[37] Kwon SR, Pallavi F, Shi Y, Oyoyo U, Mohraz A, Li Y (2018) Effect of bleaching gel viscosity on tooth whitening efficacy and pulp chamber penetration: an in vitro study. Oper Dent 43(3):326–334. 10.2341/17-099-L 29676980

[38] Torres C, Moecke SE, Mafetano A, Cornélio LF, Di Nicoló R, Borges AB (2022) Influence of Viscosity and Thickener on the Effects of Bleaching Gels. Oper Dent 47(3):E119–E130. 10.2341/20-309-L 35649221

